# Investigating the Endophyte *Actinomycetota* sp. JW0824 Strain as a Potential Bioinoculant to Enhance the Yield, Nutritive Value, and Chemical Composition of Different Cultivars of Anise (*Pimpinella anisum* L.) Seeds

**DOI:** 10.3390/biology13080553

**Published:** 2024-07-23

**Authors:** Ahmed M. Mahmoud, Ahmed M. Reyad, Maha H. Khalaf, Mohamed S. Sheteiwy, Mona F. A. Dawood, Ahmed M. El-Sawah, Enas Shaban Ahmed, Abdul Malik, Wahidah H. Al-Qahtani, Mostafa A. Abdel-Maksoud, Nermien H. S. Mousa, Mohammed Alyafei, Hamada AbdElgawad

**Affiliations:** 1 Department of Botany and Microbiology, Faculty of Science, Beni-Suef University, Beni-Suef 62511, Egyptes.ahmed@mu.edu.sa (E.S.A.); 2 Department of Integrative Agriculture, College of Agriculture and Veterinary Medicine, United Arab Emirates University, Abu Dhabi P.O. Box 15551, United Arab Emirates; salahco_2010@mans.edu.eg (M.S.S.); mohammed.s@uaeu.ac.ae (M.A.); 3 Department of Agronomy, Faculty of Agriculture, Mansoura University, Mansoura 35516, Egypt; 4 Botany and Microbiology Department, Faculty of Science, Assiut University, Assiut 71516, Egypt; mo_fa87@aun.edu.eg (M.F.A.D.);; 5 Department of Agricultural Microbiology, Faculty of Agriculture, Mansoura University, Mansoura 35516, Egypt; 6 Department of Pharmaceutics, College of Pharmacy, King Saud University, P.O. Box 270677, Riyadh 11352, Saudi Arabia; 7 Department of Food Sciences & Nutrition, College of Food and Agricultural Sciences, King Saud University, P.O. Box 270677, Riyadh 11352, Saudi Arabia; 8 Botany and Microbiology Department, College of Science, King Saud University, P.O. Box 270677, Riyadh 11352, Saudi Arabia; 9 Integrated Molecular Plant Physiology Research (IMPRES), Department of Biology, University of Antwerp, 2020 Antwerp, Belgium

**Keywords:** anise seeds, endophytes, essential oils, actinobacteria

## Abstract

**Simple Summary:**

Anise seeds offer nutritional and therapeutic benefits that are valuable to both animal and human health. This study investigated the ability of the endophytic *Actinomycetota* sp. JW0824 strain to biofortify anise seeds from Egypt, Tunisia, Syria, and Morocco. In this study, significant increases in the dry weight of seeds and oil yields were observed, along with enhancements in the levels of primary and secondary metabolites such as sugars, flavonoids, alkaloids, phenols, vitamins, and essential oils. The number of essential oil metabolic enzymes (PAL and DAHPS) was also consistently increased. The findings suggest that *Actinomycetota* sp. JW0824 could be used to enhance the yield and quality of anise seeds.

**Abstract:**

Anise (*Pimpinella anisum* L.) seeds have various nutritional and therapeutic benefits and are thus considered a valuable addition to animal and human health. Hence, in this study, we aimed to induce the nutritive and biological value of anise seeds. To this end, the potential biofortification effect of the endophytic *Actinomycetota* sp. JW0824 strain, isolated during the fall of 2023 from the medicinal plant *Achyranthes aspera*, exhibiting natural distribution in the Jazan region of Saudi Arabia, was investigated in four varieties of anise seeds from Egypt, Tunisia, Syria, and Morocco. Results revealed significant increments (*p* < 0.05) in the seed dry weight percentage (DW%) and oil yields. In line with increased biomass accumulation, the metabolism of the primary and secondary metabolites was increased. There were differential increases in proteins, sugars, flavonoids, alkaloids, phenols, vitamins (e.g., β-carotene, ascorbic acid), and essential oil components (e.g., phenylpropanoids and monoterpenes), along with their precursor phenylalanine. Consistently, the activity of L-phenylalanine aminolyase (PAL) was increased in the Egyptian and Tunisian varieties at 83.88% and 77.19%, respectively, while 3-deoxy-D-arabino-heptulosonate-7-phosphate synthase (DAHPS) activity increased in all varieties, with a significant 179.31% rise in the Egyptian variety. These findings highlight the beneficial effects of *Actinomycetota* sp. JW0824 as a bioinoculant for anise seeds, suggesting its potential application in agricultural practices to improve seed yield and quality. Further field trials are recommended to assess the commercial viability of this endophyte for enhancing anise seed production and potentially benefiting other plant species.

## 1. Introduction

Seeds are crucial for plant reproduction and preservation and functionally distribute and establish plants in different regions. The seeds of medicinal plants such as anise (*Pimpinella anisum* L.) are stores of valuable and active metabolites that are commercially and economically beneficial for medicine and pharmacy [1]. Anise seeds (aniseeds), whole or ground, have many uses and are a source of essential oils (EOs) and many extracts. Many food industries have benefited from aniseeds and their Eos, such as meat, bread, cookies, bread, biscuits, pudding, frozen dairy products, and curries, to name a few [2]. They are rich in Ca, Fe, Cu, Ka, Mn, Zn, and Mg [3]. Also, their antioxidants content and that of phytochemicals like β-carotene and vitamins A, C, and E make them good antioxidants [4]. Furthermore, they exhibit antiparasitic, digestion-stimulating, antibacterial, antifungal, and acaricidal properties [3,5,6,7]. Moreover, aniseeds have been proven to inhibit monocyte adhesion in diabetes [4] and have a potential role in glycemic control [8]. They have been used in alleviating skin aging and inflammation [9] as a source of natural estrogen [10]. Recent approaches proved the potential benefits of aniseeds in treating dysmenorrhea, diabetes, and menopausal hot flashes, as well as managing menopausal symptoms [10]. The main components of aniseed EOs are dependent on the cultivation region and extraction method. The use of aniseeds and their EOs in traditional therapies—for the relief of migraines, coughs, skin infections, congestion, and gastrointestinal distress—is attributed to trans-anethole. However, along with trans-anethole, there are other valuable components like γ himachalene, methyl chavicol or estragol, p-anisaldehyde, and eugenol. To summarize, aniseeds possess numerous applications in food, medicine, agriculture, and more. Thus, improving the quality of aniseeds increases their benefits and significance. Research has shown that the geographical origin of seeds significantly affects the nutritional composition of those seeds [2]. For example, the study conducted by Drogoudi et al. [11] revealed variations in total mineral contents and sugars among different varieties of apricot cultivars. Moreover, in a recent study, Kazi et al. [12] studied the nutritional potential of finger millet landrace seeds taken from different geographical regions. They observed that different seed varieties possessed variable nutritional compositions related to their region of origin.

While there has been research on the yield and nutritive values of anise seeds, especially focusing on essential oil composition, there are still opportunities for further exploration of the environmental and external environmental factors that can significantly impact the phytochemical profile of anise seeds. Therefore, investigating the effect of the provenance on the chemical composition of anise seeds was one of the aims of this study. Furthermore, the current research aims to explore effective approaches for sustainable agriculture, such as UV and laser irradiation, elevated CO_2_ levels, and acidic electrolyzed water, to enhance the quality of aniseeds to amplify their beneficial properties [13]. In this context, the plant-growth-promoting nature of bacteria gives them a high capacity to promote plant growth and crop yield [14,15,16,17]. Consequently, this gives actinobacteria a competitive advantage to exceed other microorganisms in the rhizosphere. Actinobacteria inoculants are effective in altering the seed-borne microbiota to promote plant traits and increase productivity [18]. Actinobacteria inoculants were reported to promote the yields of many crops, especially those of global value, such as maize, rice, and wheat [19,20]. They can improve plants by providing nutrients, increasing plant metabolites, and triggering plant resistance against rhizosphere phytopathogens such as bacteria, fungi, and others [21]. Concerning aniseeds, a few studies have demonstrated the positive impact of actinobacterial inoculants on their yield, nutrient content, and chemical composition. For instance, Darzi [22] demonstrated the positive effects on anise seed yield and nutrient composition following treatment with a combination of actinobacteria and vermicompost. Similarly, Hoseini et al. [23] demonstrated that inoculating aniseeds with *Trichoderma harzianum* and *Pseudomonas fluorescens* can boost growth under drought stress, leading to increased yield and enhanced nutrient content in the seeds. Furthermore, Sallam [24] showed that treating aniseeds with a combination of mycorrhizal fungi and actinobacteria led to the highest yield of volatile oil, total phenolic content, and total flavonoid content in dry seeds. Therefore, the application of actinobacterial inoculants might be a promising, inexpensive, and eco-friendly tool with which to decrease the harmful implications of chemical fertilizers [25].

Diverse endophytic actinobacterial species are found in various plant species, including genera such as *Streptomyces* spp., *Microbispora*, *Arthrobacter*, *Micromonospora*, etc., and novel genera such as *Phytomonospora*, *Jishengella*, *Koreibacter*, *Actinophytocola*, and others [26]. Due to their direct and intimate relationship with plant tissues, endophytic bacteria are more likely to exhibit plant-growth-promoting effects compared to bacteria in the rhizosphere [27]. The dual colonization of endophytic bacteria of both the plant rhizosphere and interior helps them stimulate plant growth more effectively than rhizospheric bacteria [28]. Endophytes are superior to rhizospheric microorganisms in disease resistance, as their internal colonization allows for continuous interaction with plant cells, enhancing the plant’s defense mechanisms [29].

Research is limited with respect to the study of endophytes like *Actinomycetota* sp. and their impact on the yield and nutritional enhancement of anise seeds at multiple growth, yield, and metabolic levels to understand the bases of bioactive primary and secondary metabolites accumulation. Thus, we aimed to investigate the positive effect of *Actinomycetota* sp. JW0824 strain as a potential bioinoculant to improve the yield, nutritive value, and chemical composition of anise seeds of four varieties from Egypt, Tunisia, Morocco, and Turkey. We hypophyses that the *Actinomycetota* sp. JW0824 strain would positively affect the yield and metabolic profile of anise seeds. Moreover, different varieties of anise seeds are expected to respond differently to the bioinoculant based on their distinct regions of origin. Thus, investigating the differential responses of the four different varieties of anise seeds to the bioinoculant will provide insight into the genetic and environmental variables. Understanding the specific biochemical mechanisms through which the endophytic *Actinomycetota* sp. JW0824 strain enhances the yield and chemical composition of anise seeds will provide insights into harnessing microbial symbiosis for agricultural sustainability.

## 2. Materials and Methods

### 2.1. Bacterial Isolation

Fresh leaves were collected from *Achyranthes aspera* in the Jazan region, washed thoroughly with running tap water and air-dried, sterilized in 70% ethanol for 3 min, followed by 0.1% mercuric chloride for 5 min, and then rinsed five times in sterilized distilled water. Sterilized small pieces were squashed, the juice was mixed with 2 mL of physiological saline solution, and about 100 microliters were plated on yeast extract mannitol agar (YEMA) and incubated at 30 °C for 3 days. The bacterial colonies were selected, sub-cultured, purified, and used for further studies, according to Devi et al. [30]. Colony morphology, shape, color, and growth pattern were recorded after 24 h of growth on YEMA. Bacterial growth at the mid-log phase was harvested via centrifugation (6000× *g*, 10 min, 24 °C), washed twice, and suspended in sterilized double distilled water. The suspension was maintained at an OD_600_ of 1.0, and the surface-sterilized seeds were inoculated in triplicates. To confirm inoculation density and purity, a culture aliquot was serially diluted and plated on a YEMA medium.

### 2.2. Molecular Identification

The method of Chen et al. [31] was used to extract genomic DNA for further gene amplification. The amplification of the 16S rRNA gene was accomplished in a PCR mix of 25 µL total volume, including PCR 1.5 mM MgCl_2_ buffer, 0.2 mM of 0.5 µM of each dNTP, a 0.625 U Taq DNA polymerase, and 5 µL of the extracted genomic DNA with primer pairs 27F (5′-CAGAGTTTGATCCTGGCT-3′) and 1492R (5′-AGGAGGTGATCCAGCCGCA-3′) [32]. The PCR temperature profile started at 95 °C for 5 min for initial denaturation, followed by 35 cycles of denaturation at 95 °C for 45 s, annealing at 49 °C for 45 s, extension at 72 °C for 90 s, and a final extension at 72 °C for 7 min. PCR products were migrated in agarose gel electrophoresis to confirm their validity. Afterward, PCR products were purified and sequenced. Finally, sequences were analyzed and compared to those in the NCBI database to identify the genus.

### 2.3. Plant Materials and Experimental Setup

Healthy and uniform seeds of six anise (*Pimpinella anisum* L.) accession cultivars, i.e., Egypt (var. Baladi), Tunisia (var. Dulce), Syria (var. Ajmer Anise-1), and Morocco (halawa2) were grown in 1.5 kg of loamy soil and organic compost (50:50%) at the soil water content (SWC) of 65%. Soils were divided into two groups, the first group inoculated with *Actinomycetota* sp. JW0824 and the second control group has no inoculation. There are four plants per pot and six pots per accession and per treatment. *Actinomycetota* sp. JW0824, was selected following preliminary screening of 9 endophytes isolated from different medicinal plants, including *Achyranthes aspera*, and 7 rhizospheric bacterial isolates from these medicinal plants’ rhizosphere. Among these, *Actinomycetota* sp. JW0824 demonstrated significant yield induction in aniseed yield and bioactive metabolites accumulation including antioxidants. Then, the pots were transferred to a controlled-growth cabinet. The growth conditions of 250 μmol PAR m^−2^ s^−1^, 22/18 °C air temperature and 60% humidity, and 16/8 h day/night photoperiod were adjusted. The plants were watered daily to stabilize the soil water content to 65% of SWC. The experiment was repeated twice. The seeds were harvested and matured (157 days after sowing). Seeds yield was estimated, and seeds were grinded in liquid N and were used for biochemical and metabolic analyses.

### 2.4. Nutrient Analyses

The total sugar content was determined as previously described by Hatanaka and Kobara [33]. Also, chloroform/methanol (2:1, *v*/*v*) was used to extract the total lipid content, following the method of Bligh and Dyer [34]. Ethanol was used to precipitate the fiber content which was then washed, weighed, and determined according to the (“Official Methods of Analysis” 1990) [35]. The total protein, total alkaloids, and total saponin contents were also measured. Ethanol (80% *v*/*v*) was used to extract flavonoids, which were then determined using the micro-plate method, with quercetin as the standard [36].

### 2.5. Vitamins Analysis

Tocopherols and ascorbate were determined through reversed-phase HPLC. Ascorbate was mixed with hexane and metaphosphoric acid in MagNALyser, respectively, and centrifuged (4 °C, 14,000× *g*, 20 min). Meanwhile, thiamine and riboflavin were separated using a reverse-phase (C18, Agilent Technologies, Santa Clara, CA, USA) column (HPLC, methanol–water) as previously described [37].

### 2.6. Determination of Total Phenolic Content

To effectively extract flavonoids and phenolics, 100 milligrams of air-dried seeds were homogenized with 1 milliliter of 80% ethanol, followed by centrifugation for 20 min at a temperature of 4 degrees Celsius. The flavonoid content was then determined using a modified aluminum chloride colorimetric method, with quercetin serving as the standard. Additionally, the total phenolic content was estimated using a Folin–Ciocalteu assay, with gallic acid acting as the reference standard [38].

### 2.7. Determination of the Levels and Metabolism of the Essential Oils

For the EO extraction, 15 g of the air-dried aniseeds was steam-distilled for 3 h in a Clevenger-type instrument. The GC/MS analysis was used to measure the concentrations (in percentage) of EOs [38]. The methodology described by Wang et al. [34] was used to determine the EO-related precursors, i.e., phenylalanine, cinnamic acid, and shikimic acid. This was conducted in an ultra-performance liquid chromatography system (Waters Acquity UPLC, Milford, Worcester County, MA, USA) coupled with a quadrupole mass spectrometer (Waters Xevo TQ, Milford, Worcester County, MA, USA) supplied with an ESI source. Also, the activities of the key enzymes in EO biosynthesis, including L-phenylalanine aminolyase, 3-deoxy-D-arabino-heptulosonate-7-phosphate synthase (DAHPS), and O-methyltransferase, were also measured, as previously described [39].

### 2.8. Essential Oil Analysis by GC-MS

GC/MS was used [40] to determine the EO contents. This was achieved through a Thermoquest GC–MS instrument (EI mode at 70 eV), equipped with a DB-1 fused silica capillary column (60 m × 25 mm id.; film thickness: 0.25 mm). The temperature was raised from 40 to 250 °C at 4 °C min^−1^ then maintained at 250 °C for 10 min, while the temperatures of the detector and injector were 300 °C and 250 °C, respectively. Helium was used as a carrier gas at a flow rate of 1.1 mL min^−1^. The identified compounds were compared to those in the NIST library based on their mass spectra.

### 2.9. Determination of DAHPS

To evaluate the DAHPS activity, we followed the method described by Wang et al. [39]. Fresh samples were homogenized in 3 mL pre-cooled Tris-HCL buffer (50 m M, pH = 7.4) containing polyvinylpyrrolidone (1%), phenylmethylsulfonyl fluoride (0.1 mM), leupeptin (10 µM), and 2-mercaptoethanol (1.4 mM). The mixture was kept at 4 °C for 30 min then centrifuged (12,000× *g*, 20 min). The supernatant was used for the assay, whereas the mixture contained the extract (0.8 mL), Tris-HCl buffer (2.2 mL, 50 mM, pH = 7.5), phosphoenolpyruvate (0.2 mM), 0.1 mM MnSO_4_/0.1 mM CoCl_2_, and erythrose-4-phosphate (0.1 mM). Afterward, it is incubated at 30 °C for 30 min. Then, the reaction was started by adding the enzyme to initiate the reaction and then terminated with 500 µL trichloroacetic acid (25%), and an enzyme-free control was used. The enzyme activity was determined by measuring the quantity of enzyme used to synthesize 1 nmol of DAHPS per minute at 30 °C. The concentration of DAHPS was then measured at 549 nm.

### 2.10. Determination of PAL

For PAL activity determination, 3 mL of precooled sodium borate buffer (0.1 M, pH = 8.8) containing polyvinylpyrrolidone (0.4%), EDTA (1 mM), 2-mercaptoethanol (5 mM) was used to homogenize the fresh samples. The mixture was kept at 4 °C for 30 min and then centrifuged (12,000× *g*, 20 min). After that, the supernatant was used for the assay; whereas the mixture contained the extract (0.8 mL), 2.2 mL sodium borate buffer (0.1 M, pH = 8.8) contained L-Phe (120 µM). The mixture was incubated at 25 °C for 40 min, the reaction was stopped by adding 120 µL HCL (6 N), and an enzyme-free control was used. The product trans-cinnamic acid was conducted at 290 nm. The deamination of 1.0 nmol of L-phenylalanine to cinnamic acid per minute was used as a basis to measure the enzyme activity.

### 2.11. Statistical Analysis

In the present study, data are represented by the mean of at least 3 replicates ± standard error. The data were initially tested for normality and homogeneity of variance through the Kolmogorov–Smirnoff and Levene’s tests using SPSS, version 20.0 (IBM Corporation, Armonk, NY, USA). To evaluate the data that meet the homoscedasticity and normality requirements, the independent samples *t*-test was utilized, while in non-parametric cases, where data did not show regularity in distribution, the Mann–Whitney U test was used. Afterward, the software Graph Pad Prism version 8.4.2 was used for plotting data, where statistically significant outcomes were defined as *p* < 0.05 and non-significant ones as *p* < 0.05.

## 3. Results and Discussion

### 3.1. Molecular Identification of the Isolate

**Figure 1:**
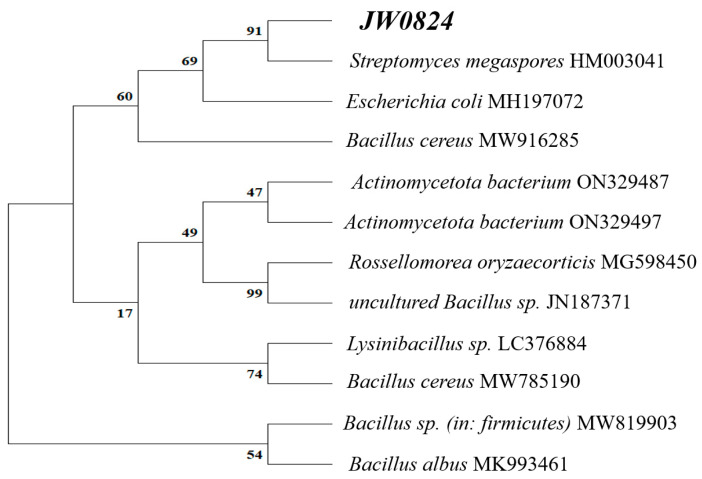
Phylogenetic analysis of the endophytic *Actinomycetota* sp JW0824. The neighbor-joining method was utilized to construct the tree based on 16S rRNA sequences. The bootstrap values for each node were determined from 100 replicates. *Actinomadura hibisca* JCM 9627 and *Escherichia coli* were used as the outgroup for this analysis.

The results of the BLAST analysis of the 16S rRNA gene sequence indicated that our isolate is closely related to Actinomycetota sp. Based on the phylogenetic tree constructed with the 16S rRNA similarity (%), it was identified as Actinomycetota sp, and clustered with Streptomyces megasporus HM003041 (Figure 1). Actinobacteria were and are still the major plant endophytes. Recent research has identified endophytic actinobacteria from a plethora of plants. For example, in the study by Mahdi et al. [41], the 16S rRNA gene sequencing revealed that all six endophytic actinobacteria isolated from Luffa cylindrica belonged to the Streptomyces genus within the phylum actinobacteria. Also, in a recent study by Maryam et al. [42], 90 endophytic bacterial isolates were obtained from the root, stem, leaf, and flowers of Anthemis pseudocotula, and 38 isolates were identified as *Actinomycetota* sp.

### 3.2. Seed Yield (Dry Weight and Water Content)

The overall results of this study showed that the *Actinomycetota* sp. JW0824 strain significantly (*p* < 0.05) enhanced almost all the investigated parameters in many of the tested aniseed cultivars, indicating the biofertilization capacity of this strain. Similarly, previous studies on different plant species have reported elevated growth parameters after inoculation with actinobacteria [43,44]. Actinobacterial inoculants were reported to enhance the plant’s ability to produce growth hormones, increase the nitrate intake, dissolve phosphate, produce essential organic compounds, oxidize sulfur, and improve root permeability, as well as to combine plant growth with bacterial exudates and thus increase the biomass and yield of both plants and seeds [45]. For instance, chickpea seeds inoculated by actinobacteria accumulated higher concentrations of Fe and Zn than untreated controls [46]. Furthermore, Villafañe [47] reported that the endophytic actinobacterial inoculant *Streptomyces* N2A increased seed yield and quality of soybeans under field conditions. Moreover, increases of 79.7% and 3% in the seed yield of *Salicornia bigelovii* and spring barley, respectively, were observed after treatment by an actinobacterial consortium over untreated controls [48,49]. Therefore, endophytic actinobacteria are among the promising tools for promoting seed yield in future agricultural practices.

**Figure 2:**
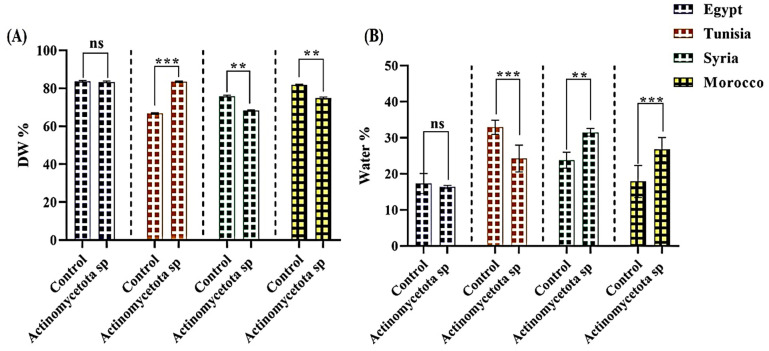
Percentage of dry weight (**A**) and water content (**B**) in control and different *Actinomycetota*-treated aniseed varieties. Data are represented by the mean of at least three replicates ± standard error. Stars (** *p* ≤ 0.01, *** *p* ≤ 0.001) above columns refer to significant differences between controls and treated seeds at *p* < 0.05. ns > 0.05 (non-significant effect).

Our results showed that treating different varieties of aniseeds with the *Actinomycetota* sp. JW0824 induced a significant increment (*p* ≤ 0.0001) in the dry weight of 24.86% and a reduction in the water content of 50.6% in seeds from Tunisia compared to their respective controls (Figure 2). However, the seed varieties from Syria and Morocco displayed significant reductions (*p* ≤ 0.0001) in the dry weight of 10.1 and 8.35%, respectively, and significant increments in the seed water contents of 32.35 and 38.34%, respectively (Figure 2).

### 3.3. The Impact on Primary and Secondary Metabolites

**Figure 3:**
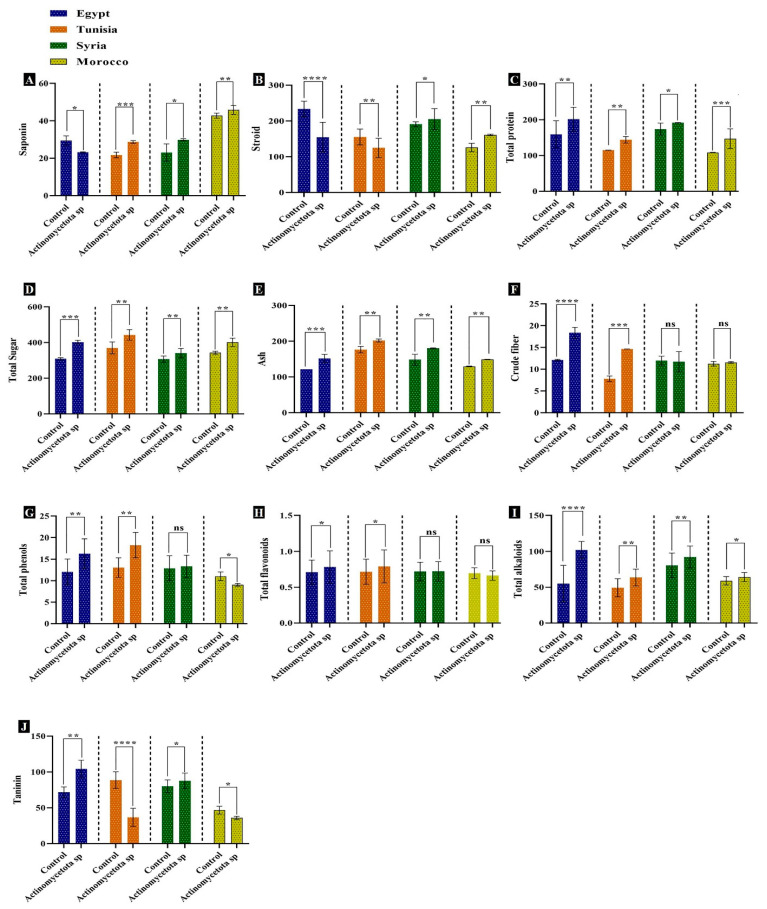
Primary and secondary metabolites in control and *Actinomycetota*-treated aniseed varieties; **A** = Saponin, **B** = steroid, **C** = total protein, **D** = total sugar, **E** = ash, **F** = crude fiber, **G** = total phenols, **H** = total falvonoids, **I** = total alkaloids, and **J** = tanins. Data are represented by the mean of at least three replicates ± standard error. Stars (*) above columns indicate significant differences between the control and the bacteria-treated samples at *p* < 0.05. * *p* ≤ 0.05, ** *p* ≤ 0.01, *** *p* ≤ 0.001, and **** *p* ≤ 0.0001. ns > 0.05 (non-significant effect).

In line with Li et al. [50], the enhanced dry weight and levels of primary and secondary metabolites in this study (Figure 3), in response to our strain treatment, could be attributed to enhanced photosynthetic activities which provide intermediates and energy for the synthesis of different metabolites. Similar results have reported improved mass gain and increased primary metabolite content in many plants like basil, peppermint, and banana [51,52]. Additionally, Sreevidya et al. [53] observed the accumulation of dry matter in chickpea seeds after enrichment of the rhizosphere with actinomycetes. Our results showed that our strain caused significant elevations in the contents of the total sugars, alkaloids, and ash (Figure 3) in all seed varieties. Treated seeds from Egypt improved levels of all metabolites and the highest increments were in crude fibers and alkaloids by 51.52 and 61.32%, respectively. Also, treated seeds from Stria and Morocco displayed significant increases (*p* ≤ 0.01) in total sugars of 10.9 and 16.94%, and in ash contents of 21.43 and 15.01%, respectively.

In this study, *Actinomycetota* sp. JW0824 increased the accumulation of primary metabolites such as the total sugar and protein contents (Figure 3), implying the accumulation of C and N contents due to the possible triggering of photosynthetic activity in these seeds. The increment of sugars improves the synthesis of many metabolites and provides the C source and energy required for the synthesis of amino acids, fatty acids, vitamins, and various secondary plant metabolites, which, in turn, promotes the nutritional quality of these seeds [54]. The increase in phenylalanine and shikimic acids, which are precursors for protein synthesis [55], also explains the increase in the total proteins in many treated seeds of this study. Furthermore, the increase in phenylalanine and shikimic acids induces the biosynthesis of secondary metabolites. In this regard, phenols and flavonoids in plants were found to be significantly influenced by actinobacterial inoculations [56]. Similarly, Singh et al. [57] reported increased total phenol contents at various stages of plant growth after the single and combined inoculation of chickpea seeds with *P. fluorescens* and *P. aeruginosa*. Also, the increments of phenolic compounds in the treated seeds in this study can be explained by the release of these compounds by our strain into the soil, their absorption by plant roots, and finally, their accumulation in plant parts, including mature seeds [58]. The elevated levels of saponin, alkaloids, phenols, and flavonoids after inoculation with our strain could add to the hypocholesterolemic potential of aniseeds. Such metabolites can provide the cell with many services, such as defense, antioxidant activity, and signaling [59].

**Figure 4:**
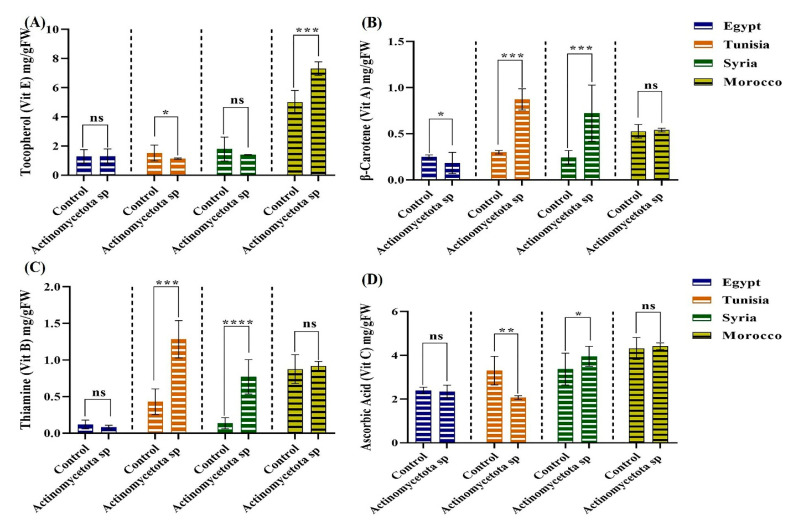
Vitamins in control and *Actinomycetota*-treated aniseed varieties. (**A**) tocopherol, (**B**) β-carotene, (**C**) thiamine and (**D**) ascorbic acid. Data are represented by the means of at least three replicates ± standard error. Stars (*) above columns indicate significant differences between the control and the bacteria-treated samples at *p* < 0.05. * *p* ≤ 0.05, ** *p* ≤ 0.01, *** *p* ≤ 0.001, **** *p* ≤ 0.0001. ns > 0.05 (non-significant effect).

For vitamins, no single seed variety showed significant increases in all vitamins (Figure 4). Also, none of the treated seed varieties showed any significant increases in β-cryptoxanthin (vitamin A) or Phylloquinone (vitamin K) compared to their untreated controls. Treated seeds from Morocco showed a significant increase of 46.2% in tocopherol (Vitamin E), while only the treated seeds of the Syrian variety showed a significant increase of 25.55% in α-carotene (vitamin A). Our strain induced significant enhancements in vitamin A (β-carotene) by 190 and 200% and in vitamin B (thiamine) by 197.67 and 450% in treated seeds from Tunisia and Syria, respectively. Additionally, only the treated seeds from Syria showed a significant increase of 17.21% in ascorbic acid (vitamin C) compared to their controls. In agreement, actinomycetes could produce and release B-type vitamins into the soil [60], which can be taken up by plant roots [61]. Similarly, Yousaf et al. [62] identified metabolites of *Acetobacter aceti*, including quinolinic acid and p-aminobenzoate, which were relevant to the induction of vitamins in barley seeds. In this study, elevated levels of vitamins in the treated seeds imply that our *Actinomycetota* strain could be a potential solution to addressing micronutrient deficiencies in the global population [63]. The outcomes of this study align with the research conducted by AbdElgawad et al. [64], who revealed increased vitamin contents (including beta and gamma tocopherols) in the seeds of soybean, kidney bean, chickpea, lentil, and pea plants that were grown in soils enriched with different isolates of actinobacteria.

The results of this study revealed a diversity in the nutritional composition of aniseeds from different geographical regions (Egypt, Morocco, Tunisia, and Syria). Similarly, previous studies showed that many factors, including geographical region and seed genotype, could significantly influence the nutritional composition of aniseeds [2]. In line with our results, Angami et al. [65] showed that different spondias species from the Eastern Himalaya varied in ascorbic acid contents, underscoring the influence of regional factors on nutritional composition. Also, González et al. [66] found that quinoa cultivars from different agroecological regions displayed variations in amino acid composition, seed yield, and total protein content. Additionally, Hinkaew et al. [67] showed that the nutritional quality of date palm fruits was influenced by multiple factors, including the growing region. Knowing the impact of geographical regions on the nutritional composition of aniseeds is important for evaluating their total nutritive quality and may have implications for their usage in food and industrial applications.

### 3.4. Improved Essential Oil Levels and Metabolism

**Figure 5:**
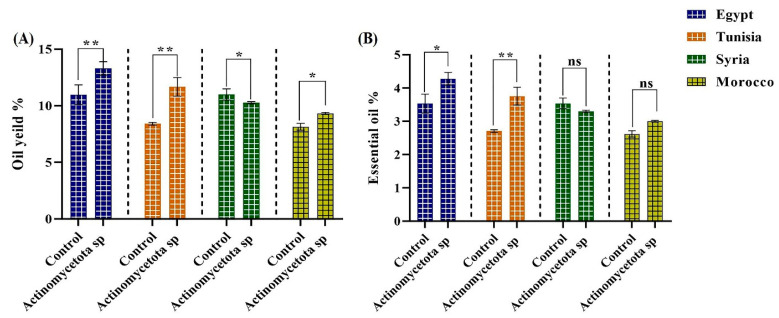
The total oil yield (**A**) and essential oil percentage (**B**) in the control and *Actinomycetota*-treated aniseed varieties. Data are represented by the mean of at least three replicates ± standard error. Stars (*) above columns indicate significant differences between the control and the bacteria-treated samples; * *p* ≤ 0.05 and ** *p* ≤ 0.01. ns > 0.05 (non-significant effect).

Previous studies reported have that the development and yield of seed and EO could be increased with bacterial inoculants in medicinal and aromatic plants such as *Pimpinella anisum* [68]. For example, *P. fluorescens* increased the total EO yield in *O. majorana* (24-fold) relative to controls [36]. Also, in another study, *P. fluorescens* induced a 50% increase (*p* = 0.02) in total essential oil yield in inoculated and co-inoculated plants [69]. The overall results (Figure 5) showed significant increases in the EOs of 21.24 and 38.74% for strains from Egypt (*p* < 0.05) and Tunisia (*p* < 0.01), respectively. Also, our results revealed that the elevated sugar contents in all the treated seed varieties (Figure 3) might serve as a precursor for synthesizing different metabolites, including essential oils, as previously reported [38].

The quality of aniseeds is determined mostly by EO content and chemical composition, and both parameters are significantly affected by environmental factors. In this study, to investigate the effect of *Actinomycetota* sp. JW0824 on the essential oil content of aniseed cultivars, the total percentage of both EO and oil yield (Figure 5), in addition to the major components (21 individual components) of EOs (Table 1), were measured in treated and untreated anise mature seed cultivars, and significant differences were observed in many cases. Our results revealed significant increments (*p* < 0.01) in trans-anethole by 42.31 and 27.50% in the treated seeds from Tunisia and Morocco, respectively. Trans-anethole was reported as the main component (90%) of aniseed essential oils. Also, trans-anethole has antimicrobial and antioxidant properties and it is responsible for the distinct aroma and taste of aniseed [70]. Also, treated seeds from Egypt, Tunisia, and Morocco showed significant increases in o-isoeugenol of 43.97, 72.27, and 11.36% and in estragole of 37.14, 46.88, and 18.24%, respectively. Also, our strain caused significant increments of 44.09, 46.46 (*p* < 0.001), and 20.72% (*p* < 0.05) in anisole levels in the varieties from Egypt, Tunisia, and Syria, respectively.

**Figure 6:**
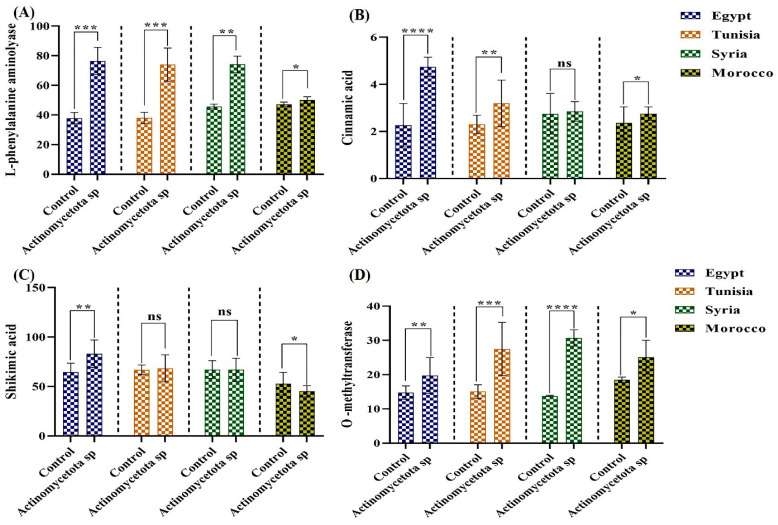
Essential oil-related precursors and related enzyme activities in the control and *Actinomycetota*-treated aniseed varieties; **A** = L-phenylalanine aminolyase, **B** = cinnamic acid, **C** = shikimic acid, and **D** = o-methyltransferase. Data are represented by the mean of at least three replicates ± standard error. Stars (*) above columns indicate significant differences between the control and the bacteria-treated samples; * *p* ≤ 0.05, ** *p* ≤ 0.01, *** *p* ≤ 0.001, and **** *p* ≤ 0.0001. ns > 0.05 (non-significant effect).

The positive impact of our strain on the amount and constituents of EOs may be attributed to the bioavailability of nitrogen and phosphate. These elements are important for EO biosynthesis and are parts of the coenzymes ATP, NADP, and CoA, which are essential for the enzymes involved in terpenoid biosynthesis [71]. Moreover, no significant impact (*p* < 0.05) on most of the investigated components of the sesquiterpene hydrocarbons except significant increases in γ-himachalene and β-Elemene by 346.66 and 85% in treated seeds from Syria, and in zingiberene by 58.06% in treated seeds from Egypt. At the metabolism level of EO, we investigated the impact of *Actinomycetota* sp. JW0824 treatment on phenylalanine, shikimic acid, cinnamic acid, DAHPS, L-phenylalanine amino-lyase, and O-methyltransferase in the tested aniseeds cultivars. Phenylpropanoids are biosynthesized via shikimic and cinnamic acids, and phenylalanine plays a major role in this process [72]. The DAHPS enzyme, 3-deoxy-D-arabinoheptulosonate-7-phosphate synthase, catalyzes the first step in the shikimic acid pathway [73]. Shikimic acid is initially converted into chorismite, which triggers the biosynthesis of phenylalanine. Phenylalanine is then converted by the phenylalanine aminolyase enzyme (PAL) into cinnamic acid, which is crucial in EO biosynthesis [39]. The increases in phenylalanine level and PAL enzyme activity (Figure 6 and Table 1) reflect the enhancement in the primary metabolism.

Our data revealed significant increases in the activity of PAL in all treated seed varieties, with the highest increases observed in seeds from Egypt and Tunisia (*p* < 0.001) by 83.88 and 77.19%, respectively (Figure 6). All the seed varieties showed a promotion in the activity of DAHPS, but only seeds from Egypt showed a significant increment of 179.31% over their untreated controls. For cinnamic acid, the most significant increments were recorded by 145.59 (*p* < 0.0001) and 38.69% (*p* < 0.01) for treated seeds from Egypt and Tunisia, respectively. Similarly, there were significant increases (*p* < 0.01) in shikimic acid of 28.53% in treated seeds from Egypt. Moreover, the O-methyltransferase activity of all cultivars was significantly increased in the treated seeds, and the highest increments were recorded for the varieties from Tunisia (*p* < 0.001) and Syria (*p* < 0.0001), i.e., 83.03 and 123.49%, respectively. Also, the enhanced enzymatic activities of both PAL and TAL (Figure 6) could be a reason for the increase in both the phenylpropanoid components (Table 1) and the flavonoid contents (Figure 3) as well [74]. In the present study, the observed increases in EO compounds may represent defensive responses to our strain. Similar increases in several EO compounds, including limonene, ocimene, etc., were reported to display insecticidal and antimicrobial effects [66]. To sum up, elevated levels of dry weight, primary and secondary metabolites such as EOs, essential amino acids, phenolic acid, and flavonoids, and an enhanced oil yield and composition among seed varieties were observable in the current study. This means that *Actinomycetota* sp. JW0824 is a good potential biofactor for improving the growth parameters of aniseeds.

## 4. Conclusions

The outcomes of this study showed that the four varieties of aniseeds from Egypt, Tunisia, Morocco, and Turkey, inoculated with *Actinomycetota* sp. JW0824, have developed higher levels of primary and secondary metabolites, including total protein, tannin, saponin, total alkaloids, essential oil, essential amino acids, phenol content, and flavonoids, than their controls. This enhances the nutritive value of food and the nutraceutical and pharmaceutical properties of aniseeds. These findings suggest that *Actinomycetota* sp. JW0824 could be beneficial for sustainable agriculture as a bioinoculant to improve the quality and productivity of aniseed cultivation. Moreover, the increased activity of precursor key enzymes of essential oil metabolism in most of the tested cultivars after treatment with our strain may promote the antioxidant and hypocholesterolemic potential of the tested aniseed varieties. However, further optimized field trials are needed before the commercial application of *Actinomycetota* sp. JW0824 for improving the growth of aniseeds or other plants.

## Figures and Tables

**Table 1:** Chemical composition of the essential oils *anise seeds* of the four varieties in response to *Actinomycetota* sp. *JW0824 inoculant*.

		Egypt		Tunisia		Syria		Morocco	
		Control	JW0824	Control	JW0824	Control	JW0824	Control	JW0824
Phenyl prostanoids	trans-anethole	75.27 ± 2.15	81.13 ± 2.20 ns	53.38 ± 1.1	75.97 ± 1.16 **	71.0 ± 0.09	61.3 ± 0.08 *	50.6 ± 2.1	64 ± 0.09 **
	o-isoeugenol	4.73 ± 3.15	6.8 ± 3.12 ****	3.21 ± 2.11	5.53 ± 2.15 ***	5.25 ± 1.11	5.26 ± 1.1 ns	3.87 ± 0.07	4.3 ± 0.05 *
	Anisole	3.22 ± 0.09	4.64 ± 1.11 ***	3.68 ± 1.17	5.39 ± 2.12 ***	3.57 ± 1.13	4.31 ± 2.15 *	3.10 ± 0.06	3.6 ± 0.0 ns
	p-anisaldehyde	0.45 ± 0.09	0.22 ± 0.001 ns	0.64 ± 0.09	0.35 ± 0.002 ns	0.30 ± 0.01	0.32 ± 0.1 ns	0.20 ± 0.07	0.24 ± 0.08 ns
	Estragole	12.68 ± 2.13	17.39 ± 1.17 **	8.51 ± 1.01	12.50 ± 2 ***	13.6 ± 1.11	13.20 ± 1 ns	9.37 ± 0.07	11.0 ± 0.09 *
Monoterpene hydrocarbons	α-pinene	0.07 ± 0.001	0.10 ± 0.003 **	0.07 ± 0.00	0.09 ± 0.001 **	61.3 ± 0.09	0.11 ± 0.0 **	0.07 ± 0.00	0.07 ± 0.00 ns
	Limonene	0.70 ± 0.13	0.75 ± 0.15 ns	0.33 ± 0.07	0.42 ± 0.06 ns	5.2 ± 0.09	0.4 ± 0.09 ns	0.25 ± 0.07	0.28 ± 0.08 ns
	Myrcene	1.53 ± 0.14	4.27 ± 1.16 ns	1.61 ± 0.08	1.90 ± 0.07 ns	4.3 ± 1.10	2.53 ± 1.1 ns	1.86 ± 0.08	1.4 ± 0.09 ns
	Linalool	2.54 ± 1.15	7.19 ± 3 ****	2.79 ± 0.06	2.74 ± 0.05 ns	0.32 ± 0.09	4.2 ± 0.00 ns	3.11 ± 0.00	2.1 ± 0.04 **
	Cis-β-ocimene	0.35 ± 0.09	0.60 ± 0.01 ns	0.36 ± 0.16	0.79 ± 0.10 ns	13.2 ± 1.10	0.55 ± 2.1 ns	0.40 ± 0.03	0.43 ± 0.06 ns
	Sabinene	0.38 ± 0.03	0.82 ± 0.19 *	0.52 ± 0.02	0.77 ± 0.01 ns	61.3 ± 1.11	0.67 ± 1.1 ns	0.48 ± 0.02	0.45 ± 0.0 ns
	p-cymene	0.53 ± 0.017	0.90 ± 0.01 **	0.97 ± 0.17	0.94 ± 0.101 ns	5.2 ± 0.00	0.86 ± 0.0 **	0.56 ± 0.08	0.46 ± 0.07 *
	Aα-phellandrene	3.37 ± 0.03	4.81 ± 0.04 **	2.42 ± 0.02	4.85 ± 0.01 ***	4.3 ± 0.08	3.14 ± 0.07 *	2.99 ± 0.09	1.9 ± 0.0 ***
Oxygenated monoterpenes	Fenchone	5.82 ± 0.04	5.74 ± 0.03 ns	5.81 ± 0.01	6.24 ± 0.02 ns	5.25 ± 0.07	5.3 ± 0.08 ns	3.65 ± 0.01	3.50 ± 0.05 ns
	1,8-cineole	2.44 ± 0.08	2.91 ± 1.10 ns	2.39 ± 1.15	2.50 ± 2.10 ns	2.43 ± 1.01	2.3 ± 2.01 ns	1.78 ± 0.07	0.9 ± 0.08 **
	α-fenchyl acetate	0.09 ± 0.04	0.15 ± 0.05 *	0.09 ± 0.02	0.17 ± 0.03 *	0.11 ± 0.06	0.25 ± 0.07 **	0.13 ± 0.05	0.12 ± 0.0 ns
	α-Terpinene	0.09 ± 0.09	0.17 ± 0.05 **	0.10 ± 0.01	0.14 ± 0.05 **	0.12 ± 0.00	0.2 ± 0.18 ***	0.10 ± 0.05	0.08 ± 0.00 *
Sesquiterpene hydrocarbons	γ-himachalene	0.12 ± 0.01	0.32 ± 0.03 ns	0.22 ± 0.02	0.13 ± 0.04 ns	0.15 ± 0.05	0.67 ± 0.07 **	0.11 ± 0.08	0.09 ± 0.0 ns
	Isolongifolene	0.38 ± 0.02	0.44 ± 0.01 ns	0.39 ± 0.03	0.44 ± 0.01 ns	0.37 ± 0.06	0.43 ± 0.0 ns	0.31 ± 0.09	0.33 ± 0.0 ns
	β-Elemene	0.65 ± 0.01	0.67 ± 0.02 ns	0.74 ± 0.01	0.66 ± 0.07 ns	0.60 ± 0.05	1.1 ± 0.01 *	0.48 ± 0.01	0.40 ± 0.04 ns
	Zingiberene	0.93 ± 0.09	1.47 ± 1.11 **	0.95 ± 0.18	1.25 ± 0.16 ns	1.09 ± 0.01	1.5 ± 0.09 ns	0.90 ± 0.07	0.96 ± 0.04 ns

Values are the mean ± standard error of three independent replicates. * indicates significant effect, where * *p* ≤ 0.05, ** *p* ≤ 0.01, *** *p* ≤ 0.001, **** *p* ≤ 0.0001, and ns > 0.05 (non-significant effect).

## Data Availability

Data are contained within the article.
